# Can Transabdominal Scan Predict a Short Cervix by Transvaginal Scan?

**DOI:** 10.1155/2017/3035718

**Published:** 2017-04-09

**Authors:** Jayaraman Mavila Nambiar, Muralidhar Vaman Pai, Arevidya Reddy, Pratap Kumar

**Affiliations:** Department of Obstetrics and Gynaecology, KMC, Manipal 576104, India

## Abstract

*Background*. To determine whether transabdominal screening can be used to screen women with short cervix on transvaginal scan.* Methods*. The study was done between 18 and 20 weeks of gestation. Transabdominal scan was done and cervical length was measured. Transvaginal scan was also done and cervical length was measured. An attempt was made to find out whether transabdominal scan be used to predict a cervical length of 25 mm by transvaginal scan.* Results*. In our study the cut-off for transabdominal scan for detecting a short cervix of 25 mm by transvaginal scan was 29 mm. A transabdominal cervical length of 29 mm could predict a short cervix of 25 mm by transvaginal scan by 100% sensitivity and 92.4% sensitivity.* Conclusion*. A cut-off of 29 mm by transabdominal scan is very accurate in predicting a short cervix of 25 mm by transvaginal scan.

## 1. Introduction

Treatment of short cervix detected by transvaginal scan by progesterones has been very effective in preventing preterm labour [1, 2]. Although cervical length was measured initially by transabdominal screening, currently the recommended method is to measure transvaginally. Transvaginal measurement of cervical length is more accurate than transabdominal measurement [3, 4]. The use of progesterone in patients with short cervix is also cost effective [3]. Incidence of short cervix in general population is extremely low requiring many women to undergo transvaginal scan [5]. Transvaginal measurement of cervical length is now a routine part of 18–20 weeks' scan. Transvaginal scan is however very uncomfortable for the patient and many refuses to undergo transvaginal scan. Some authors have suggested that transabdominal scan can predict a short cervix by transvaginal scan [6]. Some other studies have found futility of transabdominal scan in predicting a short cervix by transvaginal scan [7]. Hence, we conducted a study to find out the correlation between cervical length by transabdominal scan and transvaginal scan. Both transabdominal and transvaginal scan were done to determine the cervical length. An attempt was made to find out which cervical length by transabdominal scan that would predict a transvaginal cervical length of less than 25 mm. The main aim of study was to find out whether transabdominal scan can predict a cervical length of 25 mm by transvaginal scan.

## 2. Materials and Methods

The study was conducted as a prospective observational study in a tertiary care hospital between October 2014 and September 2016. Institutional ethical committee clearance was taken (IEC number: IEC 662/2014). Informed consent was obtained from all patients. All procedures followed were in accordance with the ethical standards of the responsible committee on human experimentation (institutional and national) and with the Helsinki Declaration of 1975, as revised in 2008. All uncomplicated singleton pregnant women between 18 and 20 weeks entered the study. Patients with multiple gestation, fetus with anomalies, and intrauterine death were excluded from the study. Cervical length was measured at the time of the scan both transabdominally and transvaginally. Philips HD 11XE machine was used for the scan. Transabdominal scan was done by 5 MHz probe and transvaginal scan was done with 8 MHz probe. In transabdominal scan a midsagittal section of cervix was obtained and cervical length was measured from internal os to external os. Patients who underwent transabdominal scan had their bladder half filled. In transvaginal scan probe was placed in anterior fornix and cervical length was measured from internal os to external os after magnifying the image. Cervical length of 25 mm by transvaginal scan was defined as short cervix. An attempt was made to correlate the cervical length by transabdominal scan which can predict a transvaginal cervical length of 25 mm. Data was analysed by SPSS 16 software. The aim of the study was to find out whether transabdominal scan predict a short cervix of 25 mm by transvaginal scan. The configuration of internal os was not considered in this study.

## 3. Results

**Figure 1:**
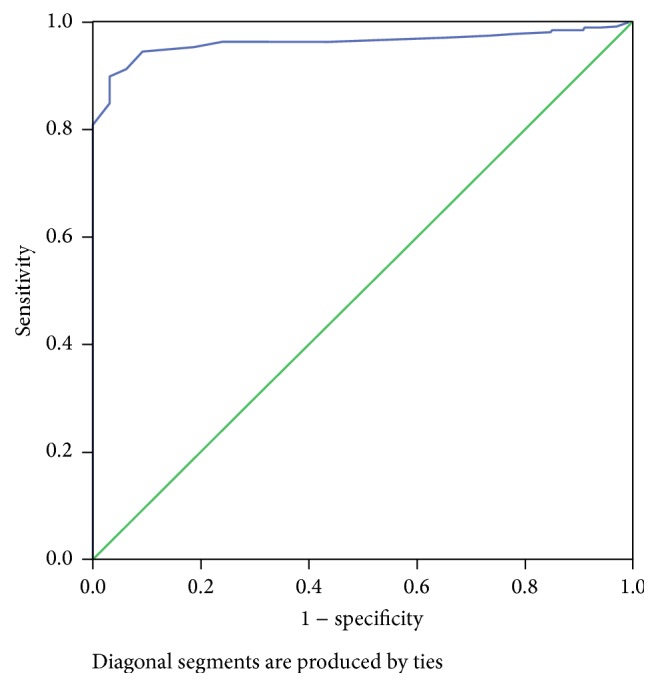
ROC curve for detecting a short cervix by transabdominal scan.

A total of 513 patients underwent scan. Out of which only 474 (92.3%) were willing to undergo both transabdominal and transvaginal scans (Table 1). The demographic characteristic of the study population is described in Table 2. Mean cervical length obtained in our study is mentioned in Table 3. The mean cervical length was shorter in both transabdominal and transvaginal group if they had preterm labour (Table 3). A cervical length of 25 mm is considered as short cervix. We constructed a ROC curve to find out which cervical length by transabdominal scan would describe a cervical length of 25 mm (Figure 1). A cervical length of 29 mm by transabdominal route would predict a cervical length of 25 mm with 100% sensitivity and 92.4% specificity.

## 4. Discussion

A cervical length of 25 mm is said to be predictive of preterm labour. However, this is done transvaginally which is most accurate for predicting preterm labour. Transvaginal scan is uncomfortable and many patients decline this scan. We in this study tried to measure cervix transabdominally and tried to find out a value which would predict a cervical length by transvaginal scan of 25 mm. We found that a cervical length measurement of 29 mm would accurately predict a cervical length of 25 mm transvaginally. We constructed a ROC curve and found that a cervical length of 29 mm by transabdominal scan was 100 percent sensitive and 94.2% specific for detecting a cervical length of 25 mm by transvaginal scan. Other studies have produced conflicting reports regarding the detection of short cervix by transvaginal scan and their correlation with transabdominal scan. We found that a value of 29 mm by transabdominal scan was 100% sensitive for detection for short cervix by transvaginal scan. Our study suggests that transabdominal screening predicts short cervix with reasonable accuracy. A cervical length was 29 mm by transabdominal scan which was highly sensitive for prediction of short cervix by transvaginal scan. We did not observe any difficulty in imaging the cervix by transabdominal scan in any patient. Thus, transabdominal screening can predict a short cervix and women can avoid a transvaginal scan if transabdominal scan shows a cervical length of more than 29 mm. Performing transvaginal scan for detecting a short cervix adds considerable time to the ultrasound examination. By doing transabdominal screening considerable time can be saved.

The study was done between 18 and 20 weeks of gestation. Further studies are needed to determine whether transabdominal cervical length is good beyond this gestation.

Stone et al. suggested that transabdominal cervical length was shorter at shorter transvaginal cervical lengths [8]. Saul et al. suggested that a 30 mm cut-off by transabdominal scan can predict a short cervix by transvaginal scan [6]. Hernandez-Andrade et al. however found that a transabdominal scan cervical length of 30 mm was relatively insensitive for predicting a short cervix by transvaginal scan [9]. Hernandez-Andrade et al. also observed difficulty in viewing the cervix transabdominally. Westerway et al. found that transabdominal screening is insensitive for detecting short cervix [7]. Hence, we may do initially do a transabdominal scan and if cervical length is more than 29 mm patient may be reassured without undergoing a transvaginal scan. Clement et al. reported that some women do find transvaginal scan unacceptable during antenatal scans [10]. Cervix can be easily visualised by transabdominal scan by most observers. Transvaginal screening of cervix however needs more training and sonographers are needed to do at least 50 scans before doing a transvaginal assessment of cervix [4, 11]. Transabdominal measurement of cervical length can have several confounding factors like bladder filling and presence of presenting part [12, 13]. We however did not observe any difficulty in observing the cervix transabdominally during our study. Transvaginal measurement of cervical length is an excellent way of measuring cervical length; however, many women refuse to undergo a transvaginal evaluation of cervix.

## 5. Conclusion

In our study a cervical length of 29 mm by transabdominal scan could accurately predict a short cervix of 25 mm by transvaginal scan. Hence, patients undergoing scan for cervical length may be screened with transabdominal scan and, if cervical length is more than 29 mm, may avoid a transvaginal scan which is uncomfortable for the pregnant woman.

## Figures and Tables

**Table 1:** Total number of scans done.

	Transabdominal scan + transvaginal scan (cases)	Declined transvaginal scan
Total number of scans done (513)	474 (92.39%)	39 (7.60%)

**Table 2:** Demographic characteristic of the study population.

Mean age of the study population	27.27 ± 4.87 years
Mean gestational age at the time of scan	19.66 ± 0.28 weeks
Mean gestational age at the time of delivery	38.01 ± 1.89 weeks
Primigravida	366
Multigravida	108

**Table 3:** Cervical length observed in our study.

		Mean cervical length (cms)	Standard deviation (cms)
Transabdominal scan	Term	3.46	0.45
	Preterm	2.58	0.48
Transvaginal scan	Term	3.15	0.45
	Preterm	2.1	0.47
